# Five U.S. Dietary Patterns and Their Relationship to Land Use, Water Use, and Greenhouse Gas Emissions: Implications for Future Food Security

**DOI:** 10.3390/nu15010215

**Published:** 2023-01-01

**Authors:** Rose Jennings, Andrew D. Henderson, Alexis Phelps, Kathryn M. Janda, Alexandra E. van den Berg

**Affiliations:** 1 Michael & Susan Dell Center for Healthy Living, UTHealth Houston School of Public Health Austin Campus, Austin, TX 77030, USA; 2 Eastern Research Group, Concord, MA 01742, USA; 3 Department of Public Health, Robbins College of Health and Human Sciences, Baylor University, Waco, TX 76706, USA

**Keywords:** dietary guidelines, dietary patterns, climate change, land use, water use, greenhouse gas emissions

## Abstract

The U.S. agri-food system is a driver of climate change and other impacts. In order to achieve environmental targets that limit global mean temperature rise ≤2 °C, a shift in American dietary patterns is critical. The purpose of this study was twofold: (1) to determine the environmental impact (i.e., land use, water use, and GHG emissions) related to consumption of five U.S. dietary patterns (i.e., Current U.S., the Healthy U.S., Mediterranean, Healthy Vegetarian, and Vegan), and (2) to determine the specific impact of each food group in each dietary pattern on the three environmental indicators. This study utilized existing datasets to synthesize information related to the study’s environmental indicators and food production and connected these data to the current U.S. diet and the USDA-defined diets. Results indicate that the three omnivore diets contributed the greatest to GHG emissions, land use and water use. The Vegan diet scored the lowest across all indicators, although the water required for plant-based protein nearly offset other water gains. For the omnivore diets, red meat and dairy milk contributed the most to each environmental indicator. By considering sustainability as well as health outcomes in their recommendations in the Dietary Guidelines, the USDA can have a critical role in shifting diets necessary to alter climate change trends.

## 1. Introduction

When all people have access to “sufficient, safe and nutritious food that meets their dietary needs and food preferences for an active and healthy life,” our world will be considered food secure [1]. Unfortunately, agri-food systems worldwide are currently facing unprecedented challenges from an increasing demand for food for a growing population, rising hunger and malnutrition, adverse climate change effects, extreme weather events, overexploitation of natural resources, loss of biodiversity, and food loss and waste [2,3,4]. These challenges can undermine the world’s capacity to meet its food needs now and in the future. To ensure food security for the current 7.6 billion as well as the estimated 83 million additional human beings being added to our planet every year, it is imperative that we examine ways to improve the sustainability of current food systems [5].

Food systems are the systems which include all the processes and infrastructure involved in feeding a population: production or growing of food, harvesting, processing, packaging, transporting, marketing, consumption, distribution and disposal of food and food-related items [6]. It also includes the inputs needed and outputs generated at each of these steps. Food systems both affect and are affected by current changes in the environment. Three main components of the environment affected by our food systems include greenhouse gas (GHG) emissions, land use and water use [7].

At a global level, it is estimated that about 34% of total greenhouse gas emissions are attributable to the global food system [8]. The GHG emissions contribution to climate change by the food system plays such a large role, that even if other industries reduced their carbon footprint, the GHG emissions by the food system could prevent meeting the Paris Climate Agreement goal of limiting global warming to 1.5 degrees C compared to pre-industrial levels [9]. 

United States (U.S.) agriculture has significant GHG emissions and use of water and land use. Agriculture and forestry together are estimated to account for 11.2 percent of GHG emissions, including methane emissions produced by raising livestock, and carbon dioxide emissions associated with agricultural energy consumption [10]. In terms of water use, agriculture uses approximately 80% of U.S. total ground and surface water [11]. Lastly, 1.5 billion acres of land in the U.S. (65%) are used for grazed forestland, pastures and ranges, and cropland [12]. These emissions and this use of resources is significant but could be more efficient, both in terms of agricultural practices and in terms of diet demand, as discussed in this manuscript. We note that the U.S. is a net exporter of food to the global market, which is facilitated by U.S. policies and resource availability [13,14].

The decreasing availability and increasing cost of water and land for agricultural use, along with increasing temperatures and intense weather patterns threaten food security for future generations [11,15,16]. Given an expected U.S. population growth of 20% by 2050 [17], land use, water use, and GHG emissions will likely continue to have a negative impact on future food availability. Taking a systems-focused approach to explore the various interconnected ways that human behaviors can be changed to mitigate the impact of climate change is necessary [18]. One potential strategy that has been explored by researchers is the role of changing dietary behaviors to reduce the contribution to climate change [19,20,21]. Specifically, the topic of adopting diets that are more sustainable, minimizing the impact of drivers of climate change, such as GHG emissions and water and land use, is warranted.

Although sustainable diets can be defined in multiple ways, the Food and Agriculture Organization (FAO) has a comprehensive definition of sustainable diets that encompasses low environmental impact, nutritional adequacy, cultural acceptability, optimization of human resources, and economic affordability [22]. It further defines sustainable diets as contributing to current and future food and nutrition security and to a healthy life for the current population while not compromising these aspects for future generations [22]. In the EAT-Lancet report published in 2019, the EAT-Lancet Commission on Food, Planet, and Health presents results from conducted an exhaustive literature review to determine the most impactful strategies for creating a sustainable food system at the global level [7]. According to this review, consuming dietary patterns that are more sustainable than the current Western dietary patterns and include less meat and dairy consumption is the most impactful strategy to increase the sustainability of our global food system [7].

### 1.1. Gaps in the Literature

While the EAT-Lancet report is based on a robust literature base on the examination of sustainable diets, much of this literature has been conducted in European countries [7,19,23]. There have only been a few studies conducted in North America focused on the U.S. food system [24]. Additionally, while the U.S.-based studies have found similar findings to those included in the EAT-Lancet report, the types of dietary behaviors and food items examined in the U.S.-based studies have been limited [20,25,26]. Lastly, many of the U.S. studies only examined one climate change indicator (i.e., GHG emissions, or water use) in isolation, rather than multiple climate change indicators simultaneously [24]. To allow for a more holistic snapshot of sustainability, it is important to examine multiple environmental outcomes simultaneously while being sensitive to the unique U.S. food system context. Given U.S.’s contribution to the global GHG emissions, which is second only to China [27], focusing on U.S. food systems is critical.

Additionally, several scholars have advocated taking a multi-level approach to assessing the role of the individual, household, and policy level when examining these issues [28]. One way to incorporate that approach is by using the Social-Ecological Model as a framework. The Social-Ecological Model proposes that in order for effective behavior change to occur, health promotion strategies must focus on the individual level, the household level, the community level or the policy level [29]. More evidence is needed in the U.S. context that (1) examines multiple indicators of environmental impact (i.e., land use, water, use, GHG emissions), (2) assess the impact of various dietary behaviors, including the U.S. dietary guidelines in the U.S. in order for true policy change to occur as sustainability guidelines are not a part of current recommendations. Having a more robust evidence base demonstrating the connection between individual-level behaviors and climate change is needed as many Americans and American politicians struggle to see the connection between individual-level behaviors and policies contributing to climate change [30,31]. 

### 1.2. Study Objective

Therefore, the overarching purpose of this study was to determine the environmental impact, defined as land use, water use, and GHG emissions of five different U.S. dietary patterns using U.S. environmental data. The five dietary patterns include the Current U.S., the Healthy U.S., Healthy Mediterranean, Healthy Vegetarian, and Vegan dietary pattern at the 2000-calorie level as defined by the United States Department of Agriculture (USDA). In addition, the specific impact of each food subgroup (e.g., red meat, green leafy vegetables) within each dietary pattern on the three environmental indicators was also examined.

## 2. Materials and Methods

### 2.1. Study Design

A cross-sectional study design that utilized existing datasets for all variables was employed to achieve the following objectives of the study: (1) collect and synthesize data related to multiple environmental burdens and food production, (2) connect these data to the current U.S. diet and the USDA-defined diets, and (3) evaluate trade-offs across environmental burdens associated with these diets. All data were sourced from national datasets or peer-reviewed literature. The scope and functionality of each dataset utilized in this study is described below. This study was approved by the Institutional Review Board of the University of Texas Health Science Center (Study No. HSC-SPH-16-0766).

### 2.2. Independent Variables

Dietary patterns that were assessed in this study were developed by the USDA to assist individuals in adhering to dietary guideline recommendations [32,33]. Since 2010, the U.S. Dietary guidelines have focused on dietary patterns as a way of capturing the reality of foods and nutrients as being consumed in various combinations over time. The 2020 Dietary Guidelines Scientific advisory committee pulled together the growing evidence that dietary pattern components “may have interactive, synergistic, and potentially cumulative relationships that can predict overall health status and disease risk more fully than can individual foods or nutrients [34].” The USDA dietary guidelines are informed by original systematic reviews; existing systematic reviews, meta-analyses, and reports by federal agencies or leading scientific organizations; data analyses; and food pattern modeling analyses [33]. The guidelines define daily amounts of foods to eat from five major food groups (i.e., Fruits, Vegetables, Grains, Proteins, and Dairy) and their subgroups (e.g., dark green vegetables, orange vegetables).

The five dietary patterns that were assessed in this study included the Current U.S. diet as well as four recommended dietary patterns as described in the 2020 versions of USDA Dietary Guidelines of Americans: Healthy U.S. (2020), Healthy Mediterranean (2020), Healthy Vegetarian (2020). Lastly, we also examined the Vegan recommended dietary pattern as described in the 2010 Dietary Guidelines as it was not included in the 2020 version [32,33]. Table 1 presents the five dietary patterns included in this study and a description of the food (sub)groups in each dietary pattern. The table also includes the serving amounts within each food (sub)group for each pattern. Consumption amounts that correspond with the Current U.S. dietary pattern were taken from the USDA Agricultural Research Service Food Pattern Equivalents Database (FPED) based on the National Health Nutrition and Examination Survey (NHANES) 2017–2018 [35,36].

Recommended amounts and limits in the USDA dietary patterns depend on 12 different calorie levels based on median length and body weight reference individuals, ranging from 1000 calories to 3200 calories per day [33]. The 2000-calorie level is considered an average, and individuals are advised to follow the dietary recommendation that meets their own needs (based on age, gender, and physical activity levels) [37]. The analyses for this study were based on a caloric level of 2000 calories for the four recommended USDA dietary patterns. The 2017 Current U.S. dietary pattern reflects the mean consumption of food groups for American adults >20 years of age in 2017–2018, who consumed on average 2155 calories [38]. For each dietary pattern, daily cups, ounces, or gram equivalents of specific foods were used as the independent variables for each analysis.

### 2.3. Dependent Variables

#### Environmental Data

For each of our three environmental indicators (i.e., land use, water use, and GHG emissions), we obtained national data sets which provided us with the environmental impact of a wide coverage of specific foods. Using the different data sets as described below, we developed a database with land use (m^2^/kg), water use (L/kg), and GHG emissions (as CO_2_ equivalents kg CO_2_ eq/kg) associated with each specific food item included in each of the five dietary patterns.

Data were taken from sources specific to each environmental variable. These sources were chosen to reflect spatial variability where possible, and to provide a wide coverage of foods. Data for directly produced commodities (e.g., vegetables or grains) are described below, followed by a discussion of how data for secondary (e.g., livestock which is fed on primary commodities) or processed (e.g., soymilk) foods are derived from primary information.

For the analysis of this study, we used a life-cycle assessment (LCA) approach. In order to deliver food to consumers, food systems go through specific cycles/stages which include the resources necessary to produce crops, feeding them to animals, processing foods, transporting them, and so forth. The data sets we used for the water and land use capture environmental inputs that occur on the farm but do not capture post-farm stages, such as processing, distributing, or use by consumers (I.e., there is some water used during processing or for washing by consumers, and some land required for the processing facility or retail). However, for water use and land use, the on-farm stage (irrigation and cultivation in the field) is by far the dominant contributor to overall life cycle resource flows [23]. In contrast, GHG emissions occur at several life cycle stages, such as transportation and refrigeration, in addition to on-farm emissions associated with fertilizer. Therefore, for GHG, it is important to consider full life cycle data, and therefore, we selected data sets that included the full life cycle data.

### 2.4. Data Sources

Land use data were taken from the U.S. Census [39], which collects and reports a variety of data related to production of various crops across U.S. states. In this case, total production and total acres harvested were used to calculate state specific values for yield and its inverse (m^2^/kg). As yield varies across the country as a function of climate, cropping practices, etc., production was used as a weight to calculate national values.

Water use data was obtained from the Pfister and Bayer data set [40]. Although the USDA NASS reports irrigation statistics in the Farm and Ranch Irrigation Survey (FRIS) [41], these data are offset from the census data by year, have reduced geographic coverage, and also have reduced crop coverage. Therefore, we used irrigation from a data set created by Pfister and Bayer [40], who modeled 160 crops at a resolution of 5 arc minutes (~10 km at mid latitudes), based on CROPWAT [42]. The importance of correctly capturing national values using weighting is illustrated using irrigation water, which varies more from state to state than does land use. For example, when calculating water consumption for corn production, an arithmetic average of state water consumption for corn yields a value of 280 L water consumed/kg corn grain. However, a production—weighted average of the same data is 61 L water consumed/kg corn grain.

As harvested crops are used as food products (e.g., fresh fruit), processed to make food products (e.g., shelling nuts, fermenting soybeans), or provided as rations to livestock, we adjusted the primary data described above using the following approaches. Conversion from wet matter to dry matter is from the FAO Global Livestock Environmental Assessment Model (GLEAM) [43]. Changes in mass due to processing (e.g., shelling nuts, preparation of flour from grain, or making non-dairy milk from nuts or soy) are taken from the World Food LCA Database (WFLDB) [44]. Crop requirements for livestock are based on the rations for North America described by GLEAM [43], and livestock dressing mass changes are also from GLEAM. Processing raw livestock products (e.g., raw milk) into secondary products (e.g., butter, cheese) are based on the WFLDB. Finally, losses up to the point of retail are based on summary data from the USDA Loss Adjusted Food Availability (LAFA) [45].

To calculate GHG emissions we used dataFIELD, a database of GHG life cycle emissions for a variety of foods [46]. This survey of GHG data associated with food production and consumption drew from a variety of LCA sources and regions. Although GHG emissions associated with food production will vary from country to country and, within the U.S., state to state, dataFIELD values are representative of foods for the purposes of national-level diet comparisons. Taking milk as an example, GHG variation is on the order of 25% across the top U.S. milk-producing regions in the U.S. [47] and indeed across most industrialized countries [48]. Thus, the region in which milk is produced is likely to have less impact on overall diet GHG than the composition of the diet itself. This observation holds for all diet components. For the purposes of this analysis, then, these GHG values can be used to show inter-diet differences.

### 2.5. Combining Environmental & Diet Data

The USDA diet data are presented in terms of food groups and food subgroups. For example, the food group vegetables, is broken down into subgroups such as leafy green vegetables, dark green vegetables, starchy vegetables, etc. In order to connect these food groups and food subgroups to the specific commodities and foods for which environmental data are available, we used the following approach.

First, subgroups were broken down to specific foods according to typical U.S. consumption patterns using the USDA’s Food Patterns and Percent Consumption (FPPC) database, which describes the consumption-based fraction that individual foods (e.g., apples, broccoli) contribute to overall food subgroups (e.g., Whole Fruit, Dark Green Vegetables) [49]. Data about U.S. consumption make it possible to convert the subgroup ‘dark green vegetables’ into specific foods and a consumption fraction, such as broccoli (35.8% of dark-green vegetable consumption), romaine lettuce (27.1%), mustard greens (1.4%). In this manner, specific foods’ environmental data were aggregated to a subgroup and group level. Note that these dis-aggregations are not specific to the diet of interest: it is thus implicitly assumed that vegetarians and omnivores eat the same fraction of specific vegetables with a given vegetable group, even if the total amounts of vegetables differ.

The Food Pattern Equivalents Database (FPED) was used to connect the food patterns to the crop data. The FPED relates masses of retail foods to the food patterns described in the diets [50]. These masses of retail foods are connected to crop and livestock commodities using the Food Intakes Converted to Retail Commodities Database (FICRCD), relates masses of retail foods to 65 retail commodities, accounting for losses during preparation [51].

We note that not all of the foods specified in the consumption patterns were reported in a given environmental inventory database. For example, the USDA Census may report data for broccoli and lettuce, but not other dark green vegetables. In priority, we looked for reasonable substitutes (e.g., lettuce may be used as a substitute for romaine lettuce). When choosing a substitute was not feasible, we used a production-weighted average of the subgroup of foods that were reported. In this way, a diet pattern was decomposed into specific food items for which substitutes or subgroup averages were available. The environmental inventory data associated with these specific food items were then summed within groups and aggregated back to the diet level.

## 3. Results

The results present the land, water, and GHG impacts for each of the five different dietary patterns. The drivers for the intra- and inter-diet trends shown in each dietary pattern are also presented.

### 3.1. Land Use

**Figure 1:**
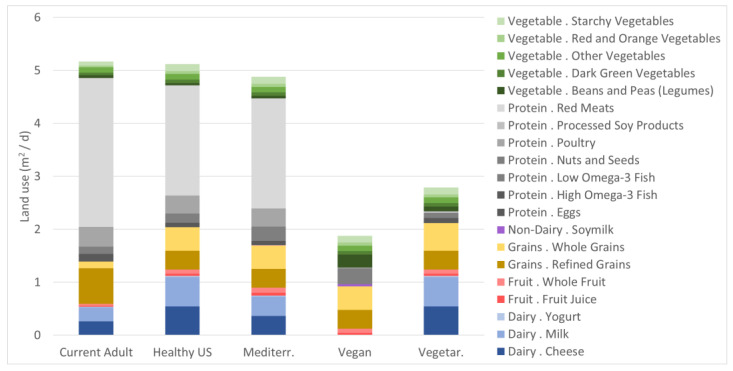
On-farm land use requirements for five diets reflecting an average of 2000 kcalories (recommended diets) or 2155 calories (Current diet), with major food group (dairy, fruit, grains, non-dairy, protein, and vegetable) and subgroups indicated by column colors.

Our results show that the omnivore dietary patterns (which include foods such as beef, pork, goat, poultry, fish, eggs, cow milk and cheese) require more land use than those that include only plant-based foods (such as fruits and vegetables, and soy milk). The Current U.S. and the Healthy U.S. dietary patterns are very closely associated with the most land use per day (5.17 m^2^ and 5.12 m^2^, respectfully) with the Healthy Mediterranean dietary pattern using slightly less land (4.88 m^2^) (Figure 1). The major contributor to land use in these dietary patterns is the red meat food group (2.08–2.81 m^2^). The amount of land used to produce red meat for each of the three omnivore diets is greater than the total amount (1.82 m^2^) of the lowest land-using dietary pattern (Vegan). Other significant food group contributors to land use in the omnivore diets examined are the dairy categories, specifically milk (0.26–0.55 m^2^) and cheese (0.26–0.55 m^2^); and grains, both refined (0.35–0.68 m^2^) and whole (0.13–0.45 m^2^). The U.S. Current diet is characterized by higher refined grained consumption therefore refined grains contribute more to land use in this dietary pattern. For the Vegan diet, refined (0.35 m^2^) and whole grains (0.45 m^2^) contribute the most to land use.

### 3.2. Water Use

**Figure 2:**
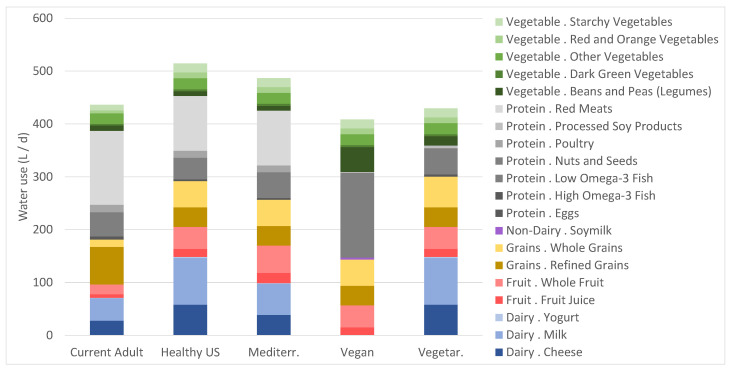
On-farm water use requirements for five diets reflecting an average of 2000 kcalories (recommended diets) or 2155 calories (Current diet), with major food group (dairy, fruit, grains, non-dairy, protein, and vegetable) and subgroups indicated by column colors.

Our study found the Healthy U.S. dietary pattern requires the most water to produce (514 L/day), followed closely by the Healthy Mediterranean dietary pattern (487 L/day) (Figure 2). The major contributors to water usage for these dietary patterns are the red meat food group followed by dairy milk. The dietary pattern which required the least amount of water is the Vegan dietary pattern (404 L/day). The nuts and seeds food group contributes 40% of total water use (161 L/day) for the Vegan dietary pattern. We analyzed water use by food group, showing that almonds follow lamb and beef as the highest water-using food groups analyzed.

### 3.3. GHG Emissions

**Figure 3:**
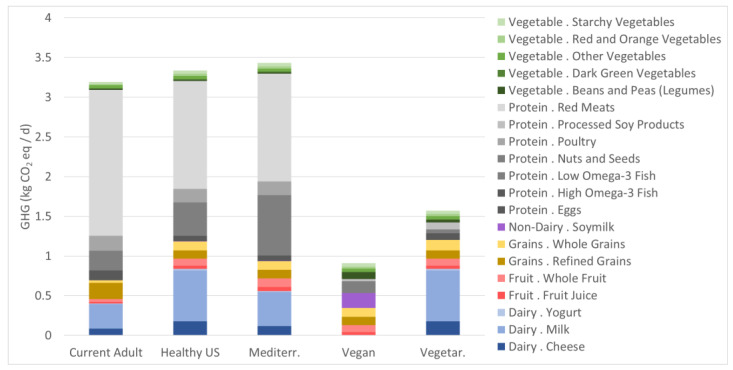
GHG life cycle emissions for five diets reflecting an average of 2000 kcalories (recommended diets) or 2155 calories (Current diet), with major food group (dairy, fruit, grains, non-dairy, protein, and vegetable) and subgroups indicated by column colors.

We found the three omnivore dietary patterns studied are the greatest contributors to GHG emissions, with the Mediterranean dietary pattern contributing to the greatest number of GHG emissions (3.42 CO_2_ eq/day) (Figure 3, followed closely by the Healthy U.S. (3.33 CO_2_ eq/day) and Current U.S. (3.19 CO_2_ eq/day) dietary patterns. The lowest GHG-emitting diet patterns are the Vegan dietary pattern (0.72 CO_2_ eq/day) followed by the Healthy Vegetarian pattern (1.57 CO_2_ eq/day). GHG emissions related to the Mediterranean dietary pattern are 4.75 times greater than the Vegan dietary pattern’s emissions. Red meat is the highest contributing food group to all the omnivore dietary patterns related GHG emissions (1.83 CO_2_ eq/day–1.36 CO_2_ eq/day), contributing 2.5 times as much GHG emissions in the Current U.S. dietary pattern compared to total GHG emissions related to the Vegan diet. Low Omega-3 fish contributed 1/5th of total GHG emissions for the Mediterranean diet. For the Healthy Vegetarian dietary pattern, 40% of related GHG emissions can be attributed to dairy milk. 

## 4. Summary and Conclusions

### 4.1. Discussion

The results from this study are congruent with findings from previous studies conducted in both Europe and the U.S, while also adding new information to the current body of literature regarding the relationship of dietary patterns and impact of indicators of sustainability.

Without having accounted for statistical uncertainty (see Limitations discussion below), our results indicate that the three omnivore diets studied have the greatest environmental impact and are related to the highest GHG emissions, land use and water use. The two vegetarian diets have the lowest impact on the environmental indicators studied, with the Vegan dietary pattern scoring the lowest for all three indicators. These findings correspond with previous studies conducted in the U.S. and internationally regarding the impact of diets on the environment [52,53]. However, these findings are important, given that they are using 2020 U.S. dietary guidelines as well as recent NHANES data, while previous studies are primarily using 2010 and 2015 U.S. dietary guidelines [20,24].

When examining the different food groups that contribute the most to GHG emissions, land use and water use, we note some distinct patterns. In the case of the three omnivore diets, the food group that contributes the greatest percentage to each of the environmental indicators in our study is red meat. Dairy milk is also of concern for all three indicators, and Low Omega-3 fish is a high contributor to GHG emissions. For the two dietary patterns that do not include animal protein, the dairy food group and the nuts/seeds food group are the food groups that score the highest for GHG emissions. For water use, the nuts/seeds group are the largest contributors for the Vegan pattern, while dairy milk is the largest contributor in the Vegetarian diet. Lastly, for land use, for the Vegetarian diet, dairy milk, dairy cheese, and whole grains are the largest contributors while for the Vegan diet, whole grains and refined grains are the largest contributors. However, the food groups that are the largest contributors to the three environmental indicators in the Vegetarian and Vegan diets contribute much less to GHG emissions, land use and water use than the food groups that are the highest contributors to the omnivore dietary patterns.

Previous studies have found similar results with red meat/beef being the highest contributor to several critical environmental indicators [54]. As energy is lost at each trophic level, the production of meat is less efficient and consequently produces more GHG emissions per unit of energy compared to plants [55]. Red meat from ruminants has high levels of methane emissions caused by the decomposition of their manure under certain conditions, and by enteric emissions. Although methane, which has a low half-life in the environment compared to CO_2_ eq, it has a relatively high warming potential and is considered more detrimental to the environment than CO_2_ eq.

Our findings suggest that the most impactful diet-related change that Americans can make towards a more sustainable diet is a shift towards a Vegetarian or Vegan diet and reducing the consumption of red meat and potentially dairy products [56]. These key takeaways are in line with previous studies that found that shifting from an average meat containing diet to a vegetarian diet would require anywhere from 18–50% lower GHG emissions, or 23–31% less GHG emissions in the case of a vegan diet [57,58]. The EAT Lancet Commission report, which may be considered the most exhaustive review of studies related to this topic, recommends a diet that reduces meat consumption by 50% for individuals living in the U.S. [7]. Although such a shift in dietary behaviors may not seem feasible for many Americans, a recent study found that from 2003–2018, the mean GHG emissions associated with the U.S. diet reduced by approximately 35%, from 4.02 kg CO_2_ eq per day per capita, to 2.45 kg CO_2_ eq per day per capita, and average beef consumption declined 40% per capita, which contributed to more than 50% of the observed GHG savings in the diet over the study period [59]. Men aged 20–34 had the greatest decrease in rate of reduction in GHGs associated with diet changes, while Black women had the lowest GHG emissions associated with their diet [59]. However, even with this substantial decrease in GHG emissions due to dietary change, Americans are still exceeding established GHG limits to meet global targets and additional efforts to decrease diet related GHG emissions are warranted.

Interestingly, the diet which is recommended by the U.S. Dietary Guidelines for all Americans to follow (i.e., the Healthy U.S.) does not recommend a substantial decrease in red meat. As a result, the Healthy U.S. dietary pattern has a greater impact on GHG emissions and land use than the Current dietary pattern. These results are congruent with several other studies examining the potential negative environmental impacts from switching from the current to a USDA recommended omnivore diet [25,60,61]. Given the importance of consuming diets that are sustainable, this recommendation seems to contrast with what Americans should be consuming.

Americans and American politicians struggle to see the connection between individual-level behaviors and policies contributing to climate change [30]. Perhaps this study provides the evidence needed to clearly establish the association of individual-level diet and climate change in the U.S. context. A diet which includes less red meat and more plants is not only beneficial for sustainability, but it can also have a major impact on the health of individuals, although each person’s nutritional status must be taken into account [9,62,63]. A recent cohort study found that dietary patterns that were associated with less cardiovascular risk were also associated with less GHG emissions, lower fertilizer, cropland and water needs [64]. Given the impact of overconsumption of red meat on both the environment and the health of Americans, this underscores the need to encourage Americans to consume less red meat.

According to the Socio-Ecological Model [29], potential strategies to change behaviors can focus on the individual level, the household level, the community level or the policy level. Given the scale of influence of the Dietary Guidelines for Americans on food systems, incorporating sustainability into the development of the next set of Dietary Guidelines has the potential to have great benefit in terms of long-term food security [60]. Other countries have been able to do this successfully, such as Canada, Sweden, Denmark, Spain and Switzerland, and can serve as example to the U.S. [65,66,67]. Other policy strategies that may help reduce the overconsumption of red meat in the U.S. include economic policies (i.e., taxing red meat and decreasing cost of high omega-3 fish and fruits and vegetables) and agricultural policies and regulations (i.e., shifting subsidies from factory farms to smaller farms, voluntary and non-voluntary mitigation strategies for livestock farming) [68].

Past studies suggest that individual dietary behavior change can best be accomplished through policy change, however actions by governments must be paired by societal support to be effective [69]. A recent review of environmental sustainability in national food-based dietary guidelines found that only a handful of these national guidelines that include statements about environmental sustainability provide specific advice about to implement these guidelines [70]. Strategies focused on the individual and household level may include creative health promotion via social media campaigns that aim to increase willingness to shift towards plant-based eating by raising awareness of the detrimental health and planetary effects of red meat, and showing that alternatives can be both healthy, tasty and cost effective [71,72]. These campaigns could also be incorporated into the educational component of household-targeted U.S. food assistance programs such as Women, Infants and Children (WIC) and the Supplemental Assistance Nutrition Program (SNAP), paired with alternative offerings to standard food packages (i.e., alternative milk and meat) without special dietary restrictions required. Strategies focused on the community level include social marketing campaigns or promoting these climate-friendly dietary shifts in schools, universities, government-subsidized programs, and via meal programs in larger corporations. Public health nutrition researchers and practitioners will be key instruments in accomplishing this societal change needed in order to achieve greater policy change [66,72].

Additionally, future research opportunities exist to ask these questions we have asked in lower and middle-income countries, where persisting research gaps have been identified [73] and a need to incorporate environmental sustainability in national food-based dietary guidelines in these countries [70].

### 4.2. Limitations

There are certain limitations to the approaches utilized in the analyses for this study. The land and water use data in this study captures only water and land used in farm production and not post-farm transportation, manufacturing, etc. Water use for animal production, such as beef, does not include the water consumed by animals. However, the datasets we utilized were the most comprehensive data sets available. The lack of available data sets highlights the need for data sets that also include required land and water use for processing, manufacturing, and distribution of individual foods. A second limitation is that the agricultural data are based on national averages that consider regional variation but are not differentiated by agricultural practice (e.g., organic vs. conventional production), which may vary in water, land, and machinery usage. At this time, however, organic practices are not representative of the entire country, as certified organic cropland and pasture accounted for only around 1% of the U.S. total farmland in 2011 [74].

We also did not take into account the variation between diets in different states, nor did we conduct a sensitivity analysis for the surrogates/proxies that we chose for the food items. Although these differentiated analyses would provide data for an interesting and useful future study, they require data that currently is not available.

Lastly, another limitation is that GHG emissions, water use and land use related to the management of food waste were not considered in this analysis. Without taking waste into account, our analysis underestimates the true environmental impact for all foods.

Many of our errors should be systematic across diets and, thus, are expected to have a minimal impact. Therefore, relative trends from our results, rather than absolute numbers or absolute differences, should be emphasized. An analysis that includes statistical precision and error has not been conducted and is an area for future research. Without having examined statistical uncertainty, all assertions about relative benefits or impacts of different diets are based on point estimates and should be considered interim conclusions.

### 4.3. Policy Implications

This study is a unique environmental assessment of U.S. dietary patterns, as it uses U.S. data and incorporates multiple environmental variables simultaneously. The results provide more environmental data, beyond GHGs, to facilitate a deeper reflection on the potential trade-offs of dietary patterns. The findings indicate that by switching to dietary patterns characterized by less red meat, such as the Vegan and Healthy Vegetarian-Style dietary patterns, less land use, water use, and GHG emissions will be the per-capita result for food production, as based on current practices. These implications call for an examination of agricultural practices and policy and policies in schools and communities as well as creative health promotion strategies to reduce barriers and increase consumption of foods that are more environmentally friendly (e.g., cooking classes, increasing SNAP/WIC usage, reducing the cost of fruits and vegetables).

There are many environmental, economic, and social externalities that were not included in this analysis but would add to a more holistic snapshot of the sustainability of diets. To meet the FAO’s definition of sustainable diets, nutritional adequacy, cultural acceptability, optimization of human resources, and economic affordability also need to be considered [75]. There is potential for a greater breadth of sustainability indicators to be included in food-based dietary guidelines [70]. Currently, data for these types of indicators in the U.S. are limited. More studies that include primary data and nationally representative samples are warranted to begin to understand how these indicators also may contribute to the sustainability of our diets.

## 5. Conclusions

This study has important policy and health promotion implications. Shifting towards more plant-based diets could result in reduced environmental impact. Reduced water, land use and GHG emissions could improve household food security in the U.S. and global food security for a growing population. Continued improvement in production methods for animal-sourced proteins, namely red meat, dairy milk and cheeses and Low omega-3 fish will be of interest. However, improving production methods for plant-based proteins, e.g., water use for almonds, could also have positive implications for creating more sustainable diets, thus greater food security for the planet. More work is needed in understanding how to encourage individuals to adopt sustainable dietary behaviors, such as more plant-based diets, to foster greater household food security in the U.S. and globally.

## Figures and Tables

**Table 1:** Dietary Pattern Comparison: Current U.S., Healthy U.S., Mediterranean, Vegetarian, and Vegan Food Patterns; USDA diet pattern quantities reflect recommendations for a 2000 kcal level diet. Values are servings (units indicated by dietary item, first column) per day.

Pattern	Current U.S.	Healthy U.S.	Mediterranean	Vegetarian	Vegan
Source	NHANES (2017–2018)	DGA 2020–2025	DGA 2020–2025	DGA 2020–2025	DGA 2010–2015
	Includes mean consumption for American adults >20 years old. Characterized by high meat and refined grains intake, and low in dairy, fruits, vegetables, whole grains, nuts, seeds, and seafood.	Omnivore diet that includes more fruits, vegetables, whole grains, dairy, nuts, and seafood and less refined grains and meat than the current U.S. diet pattern.	Omnivore diet that includes slightly more seafood and less dairy than the U.S. Healthy Diet pattern.	Vegetarian diet pattern that excludes meat, poultry, and seafood, and includes more eggs, legumes, nuts, seeds, and soy than the U.S. Healthy Diet pattern.	Vegetarian diet pattern that excludes all animal protein and includes more legumes, nuts, seeds, and soy than the Vegetarian Pattern. Milk and milk products group includes non-dairy (soy milk).
Vegetables: total (c)	1.55	2.52	2.52	2.74 *	4.40 *
Dark green (c)	0.16	0.21	0.21	0.21	0.21
Beans and peas (c)	0.12	0.21	0.21	0.43	0.68
Red and orange (c)	0.38	0.79	0.79	0.79	0.79
Other (c)	0.56	0.57	0.57	0.57	0.57
Starchy (c)	0.45	0.71	0.71	0.71	0.71
Fruit and juices (c)	0.84	2.00	2.00	2.00	2.00
Grains: total (oz)	6.64	6.00	6.50	6.00	6.00
Refined grains (oz)	5.76	3.00	3.00	3.00	3.00
Whole grains (oz)	0.84	3.00	3.50	3.00	3.00
Milk and milk products (dairy products): total (c)	1.44 **	3.00	2.00	3.00	3.00 **
Protein foods: total (oz)	6.33	5.61	6.55	3.43	5.43
Meat (oz)	2.60	1.80	1.80	0.00	0.00
Poultry (oz)	1.58	1.50	1.50	0.00	0.00
Eggs (oz)	0.62	0.40	0.40	0.43	0.00
Fish/seafood (oz)	0.62	1.20	2.14	0.00	0.00
Legumes (beans/peas) (oz)	0.48	--	--	0.86 *	1.86 *
Nuts, seeds, and soy products (oz)	0.91	0.71	0.71	2.14	3.57

Notes. Serving units are “c”, cups (as volume), and “oz”, ounces (as mass). * Additional serving recommendations of legumes/peas for the protein group are added to the total vegetable serving sizes for the Vegetarian and Vegan Diet Patterns. ** Includes Non-dairy calcium fortified (c).

## Data Availability

Data can be made available by request by contacting the authors.
